# Understanding the interactions between the bis(trifluoromethylsulfonyl)imide anion and absorbed CO_2_ using X-ray diffraction analysis of a soft crystal surrogate

**DOI:** 10.1038/s42004-020-00390-1

**Published:** 2020-10-27

**Authors:** Xin Zheng, Katsuo Fukuhara, Yuh Hijikata, Jenny Pirillo, Hiroyasu Sato, Kiyonori Takahashi, Shin-ichiro Noro, Takayoshi Nakamura

**Affiliations:** 1 Graduate School of Environmental Science, Hokkaido University, Sapporo, 060-0810 Japan; 2 Institute for Chemical Reaction Design and Discovery (WPI-ICReDD), Hokkaido University, Sapporo, 001-0021 Japan; 3 Rigaku Corporation, Akishima, Tokyo, 196-8666 Japan; 4 Research Institute for Electronic Science, Hokkaido University, Sapporo, 010-0020 Japan; 5 Faculty of Environmental Earth Science, Hokkaido University, Sapporo, 060-0810 Japan

**Keywords:** Soft materials, Coordination chemistry, X-ray diffraction

## Abstract

The selective carbon dioxide (CO_2_) absorption properties of ionic liquids (ILs) are highly pertinent to the development of methods to capture CO_2_. Although it has been reported that fluorinated components give ILs enhanced CO_2_ solubilities, it has been challenging to gain a deep understanding of the interactions occurring between ILs and CO_2_. In this investigation, we have utilized the soft crystalline material [Cu(NTf_2_)_2_(bpp)_2_] (NTf_2_^‒^ = bis(trifluoromethylsulfonyl)imide, bpp = 1,3-bis-(4-pyridyl)propane) as a surrogate for single-crystal X-ray diffraction analysis to visualize interactions occurring between CO_2_ and NTf_2_^‒^, the fluorinated IL component that is responsible for high CO_2_ solubility. Analysis of the structure of a CO_2_-loaded crystal reveals that CO_2_ interacts with both fluorine and oxygen atoms of NTf_2_^‒^ anions in a *trans* rather than *cis* conformation about the S–N bond. Theoretical analysis of the structure of the CO_2_-loaded crystal indicates that dispersion and electrostatic interactions exist between CO_2_ and the framework. The overall results provide important insight into understanding and improving the CO_2_ absorption properties of ILs.

## Abstract (web summary)

The selective CO_2_ absorption properties of ionic liquids make them attractive for CO_2_ capture purposes, but their tendency to crystallize poorly hampers structural investigations. Here a soft crystalline material is used as a surrogate for single-crystal X-ray diffraction analysis to visualize interactions between CO_2_ and NTf_2_^−^, the fluorinated component that is responsible for high CO_2_ solubility.

## Introduction

Ionic liquids (ILs) have received increasing attention in the past several decades owing to their wide variety of potential industrial applications^1–3^. This is especially true for ILs that have selective carbon dioxide (CO_2_) absorption properties as a result of the tremendous interest in and urgency for stemming global warming by removal of green house gases from the atmosphere^4^. It is known that anion components of ILs significantly influence CO_2_ absorption capacities and that fluorine containing anions are superior CO_2_ absorbers as compared to those that lack this halogen^5^. This phenomenon is exemplified by the bis(trifluoromethylsulfonyl)imide (NTf_2_^‒^) anion, which is one of the most interesting building blocks for the construction of ILs with high CO_2_ absorption propensities^6^. Although several theories have been advanced to explain the physical interactions that take place between NTf_2_^‒^-containing ILs and CO_2_, some of which have been tested experimentally and by using simulations^7–12^, a full understanding of the interactions has not yet been gained. In particular, based on the current state of knowledge, it is still not possible to ascertain whether oxygen or fluorine is the key CO_2_ absorption site in NTf_2_^‒^, and to determine the nature of primary interactions occurring between this anion and this gas. The main hurdle to obtaining this information is associated with difficulties with determining the structures of ILs owing to their non-crystalline nature. In addition, the conformationally flexible structure of the NTf_2_^‒^ anion, originating from reasonably rapid rotation (barrier of 25.1 kJ mol^−1^) of the CF_3_SO_2_ substituent around the S–N bond (Supplementary Fig. 13), complicates elucidation of the IL-CO_2_ interactions.

Recently, a new class of materials, referred to as soft crystals, has attracted great attention. These materials can exist in both a highly crystalline and soft states, which can be reversibly interconverted in response to external chemical and physical stimuli^13,14^. Interestingly, some of the soft materials retain their single crystalline nature upon being treated with external stimuli. We envisaged that it might be possible to design and fabricate soft crystals, which contain components that are the same as or similar to those present in ILs, and that might possess the ability to absorb CO_2_ in synchrony with stimuli promoted structural changes. In this way, it would be possible to utilize these substances as surrogates to determine the structures of and elucidate important interactions in ILs containing absorbed CO_2_ using standard single-crystal X-ray diffraction techniques.

**Fig. 1:**
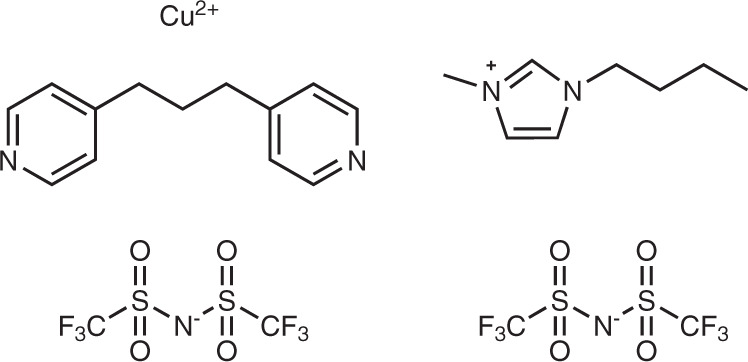
Molecular structures. Structures of the components of soft crystal **1** (left) and a typical IL [1-*n*-butyl-3-methylimidazolium][NTf_2_^‒^] containing NTf_2_^‒^ (right)^6^.

To explore this proposal, we prepared a new soft crystal [Cu(NTf_2_)_2_(bpp)_2_] (**1**), which is composed of an NTf_2_^‒^ anion and the flexible 1,3-bis(4-pyridyl)propane (bpp) ligand and Cu^2+^ (Fig. 1) that possess an alkyl chain and positively charged Cu^2+^ coordinated nitrogen centers that are typical of those present in a ILs (e.g., the alkylimidazolium and NTf_2_^‒^ containing IL in Fig. 1). In the effort described below, we demonstrated that the soft crystal **1** displays reversible absorption of CO_2_ associated with a stimulus-induced structural transformation, while retaining its permanent single-crystalline nature. Moreover, by using standard single-crystal X-ray diffraction techniques, we elucidated the nature of interactions with the NTf_2_^‒^ anion that are responsible for CO_2_ absorption.

## Results

### Crystal structure of as-synthesized soft crystal

**Fig. 2:**
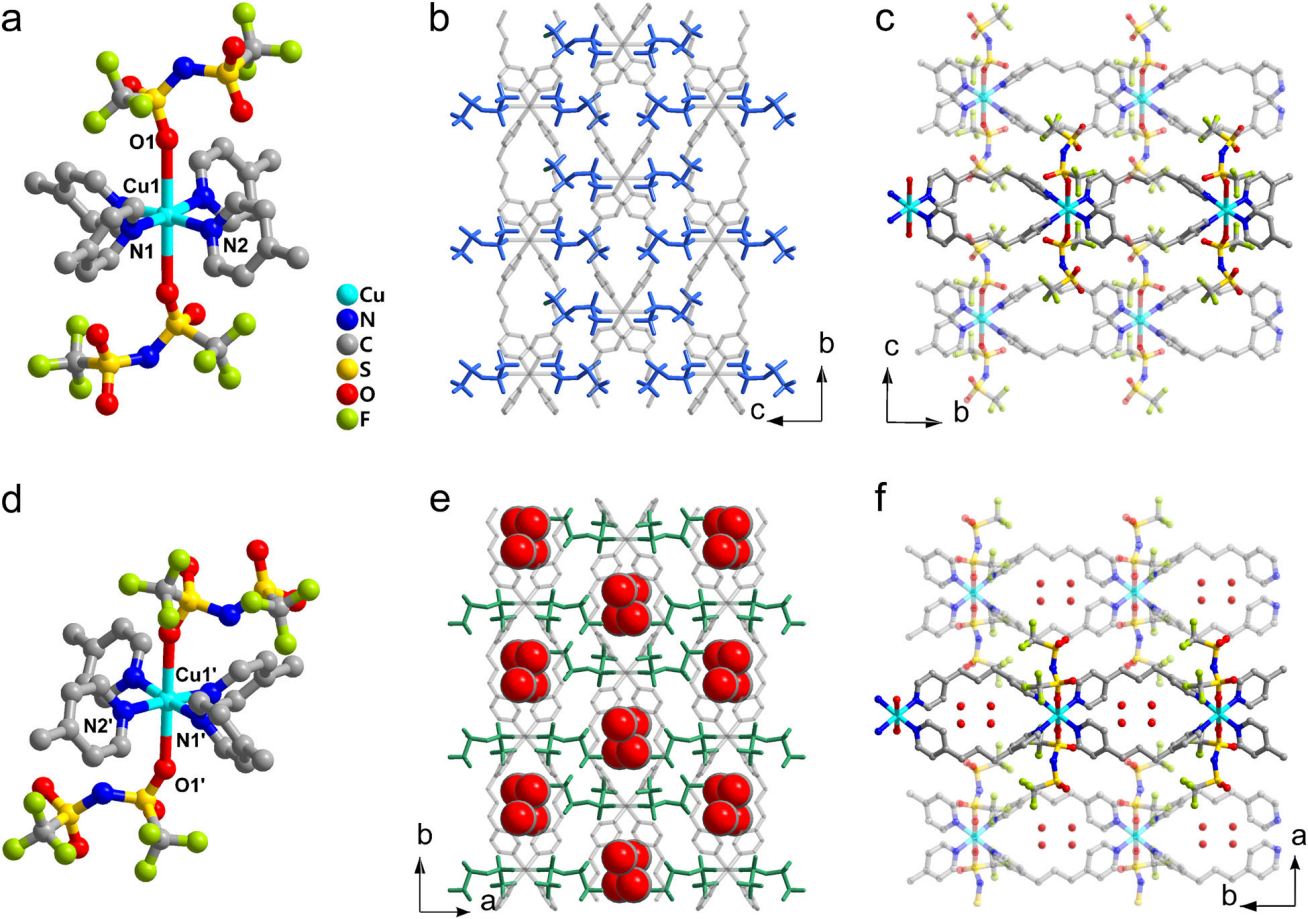
Crystal structures. Views of the crystal structures of **a**–**c** **1** and **d**–**f** **1**·2CO_2_. Coordination environment around the Cu center in **a** **1** and **d** **1**·2CO_2_. Conformation of NTf_2_^‒^ in **b** **1** and **e** **1**·2CO_2_. 1D chain structures in **c** **1** and **f** **1**·2CO_2_.

A single-crystal of **1** was prepared by using the diffusion method and a three-phase system, including a 0.1 M aqueous Cu(NTf_2_)_2_ bottom layer, a 1:1 water and MeOH, middle layer, and 0.2 M bpp in MeOH top layer. Upon standing, this system generated transparent and rod-like purple single crystals, which were shown by using the elemental analysis to have the desired atomic composition and purity. The soft crystal **1** crystallized in the monoclinic space group *P*2/*n*, containing crystallographically independent one copper ion, two NTf_2_^‒^ anions and two bpp ligands. The copper ion, surrounded by two NTf_2_^‒^ anions and four bpp ligands, exists in a distorted octahedral environment (Fig. 2a). The distance between the copper ion and NTf_2_^‒^ anions (Cu1-O1 = 2.801(2) Å) is much longer than that between the copper ion and the bpp ligands (Cu1–N1 = 2.002(2) Å, Cu1–N2 = 2.013(3) Å) as a consequence of Jahn-Teller distortion and the weak coordination ability of NTf_2_^‒^. The NTf_2_^‒^ anions exist in a *trans* conformation (Fig. 2b and Supplementary Fig. 1), which is known to be thermodynamically more stable than the *cis* conformation^15^. The bpp ligands bridge the copper ion to form one-dimensional (1D) chains oriented along the *b*-axis with a toroidal space of 6.4 Å × 12.2 Å (Fig. 2c and Supplementary Fig. 2). The 1D chains aggregate through weak CH/π interactions to form quasi two-dimensional (2D) layers with an interchain distance of 9.9 Å (Supplementary Fig. 3). In addition, NTf_2_^‒^ anions are sandwiched between the 2D layers through weak coordinative interactions, in which the distance between two neighboring layers is 8.0 Å (Supplementary Fig. 3). The 2D layers and NTf_2_^‒^ anions are densely packed through the above-mentioned weak coordinative interactions and weak hydrogen bonding interactions between the trifluoromethylsulfonyl groups of NTf_2_^‒^ and pyridyl groups of the bpp ligands (Supplementary Fig. 4). As a result, almost no pores exist in the crystal structure of **1**, and the pore space calculated by using MERCURY software (probe radius: 1.2 Å, approximately grid spacing: 0.7 Å) is only 0.7%.

### CO_2_ absorption properties and stability for water

**Fig. 3:**
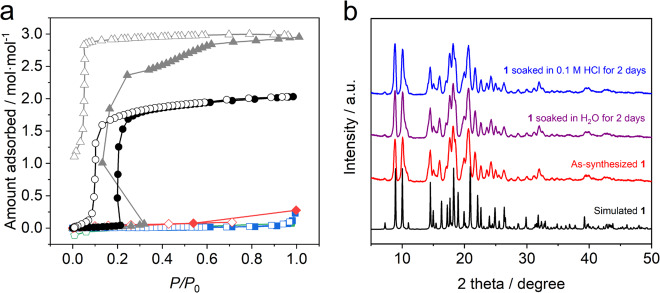
Absorption/desorption properties and PXRD patterns. **a** Absorption (closed symbols)/desorption (open symbols) isotherms of CO_2_ (black circle), N_2_ (blue square), Ar (green pentagon), and H_2_O (red rhombus) at 195 K (CO_2_), 77 K (N_2_ and Ar), and 298 K (H_2_O), respectively, for **1** and of CO_2_ (gray triangle) at 195 K for **2**. **b** Calculated PXRD pattern of **1** from its single-crystal X-ray structure (black) and observed PXRD patterns of as-synthesized **1** (red), **1** soaked in H_2_O (purple) and 0.1 M HCl (blue) for 2 days.

Inspection of absorption/desorption isotherms of **1** displayed in Fig. 3a shows that almost no absorption of N_2_ and Ar occurs at 77 K proving that this soft crystal has a densely assembled structure. In contrast, **1** exhibits a steep increase and decrease in the amount of absorbed CO_2_ at *P*/*P*_0_ = 0.21 and 0.09, respectively, and 195 K, while N_2_ and Ar are not absorbed even at 195 K (Supplementary Fig. 9), which suggests that absorption of this gas takes place in association with a change between a closed and an open structure^16^. The saturated absorption amount of CO_2_ was found to be 2.03 mol mol^−1^ at *P*/*P*_0_ = 0.98 (*P* ≅ fugacity = 0.99 bar), which corresponds to 1.02 mol per mol of NTf_2_^‒^. This value is close to those of NTf_2_^‒^-containing CO_2_ absorbing ILs at 313 K and 40 bar (fugacity = 34 bar) (under this condition, absorption by NTf_2_^‒^-containing ILs is ca. 0.5 CO_2_ mole fraction, corresponding to 1 mol per one mol of NTf_2_^‒^)^17^. In order to elucidate the role of fluorinated moieties in governing CO_2_ absorption, we synthesized the nonfluorinated analog of [Cu(NMes_2_)_2_(bpp)_2_] (**2**), in which NMes_2_^‒^ is bis(methylsulfonyl)imide, which has a one-dimensional structure that is similar to that of **1** (Supplementary Fig. 6) and measured its absorption properties. As shown in Fig. 3a and Supplementary Fig. 10, CO_2_ is selectively absorbed to **2** but the threshold pressure in the absorption isotherm (*P*/*P*_0_ = 0.32) is higher than that of **1** (*P*/*P*_0_ = 0.21), indicating that the fluorinated component facilitates the CO_2_ absorption. A similar observation has been made in studies of the ILs with NTf_2_^‒^ and NMes_2_^‒^ anions^5^. The integer number (2 mol mol^−1^) of the amount of CO_2_ absorbed at saturation may be related to the free volume and/or specific absorption sites in the soft crystal **1**.

The results of investigation of the variable-temperature CO_2_ absorption of **1** reveal that similar behavior is followed but that different threshold pressures exist in the absorption and desorption isotherms (Supplementary Fig. 11). Because the threshold positions are related to equilibrium pressures for the CO_2_ absorption reaction, their temperature dependence enables an evaluation of the enthalpy of CO_2_ absorption (*Q*_st_) by using the Clausius-Clapeyron equation. A plot of the natural logarithm of the threshold positions vs 1/*T* was found to be linear (Supplementary Fig. 12), and analysis of the slope yields a *Q*_st_ value of ‒29.1 kJ mol^−1^. This enthalpy value is only moderate when compared with those of other CO_2_ absorption/adsorption materials, including a 30 wt% monoethanolamine solution (‒72 to ‒79 kJ mol^−1^)^18^, amine-functionalized porous organic polymer (‒61 kJ/mol)^19^ and zeolites (‒49.1, ‒50.0, and −38.0 kJ mol^−1^ for NaX, Na-ZSM-5 and H-ZSM-5, respectively)^20^. [1-*n*-Alkyl-3-methylimidazolium][NTf_2_^‒^] (alkyl = ethyl, butyl, and hexyl) ILs have smaller *Q*_st_ values ranging from ‒11 to ‒14 kJ mol^−1^ because of more limited physisorption between dynamic liquid and a gas phase^21^. The only moderate *Q*_st_ value among those of other solid adsorbents suggests that the interaction between NTf_2_^‒^ in **1** and CO_2_ and/or that between bpp and CO_2_ is not strong.

The stability and absorbability of **1** for water, which are important issues when contemplating practical uses, were also investigated. NTf_2_^‒^ anion is hydrophobic as reflected by the fact that the IL [1-*n*-butyl-3-methylimidazolium][NTf_2_^‒^] (Fig. 1) has a low absorbability for water and a CO_2_ absorption capacity that is not affected by water absorption^17^. The soft crystal **1** also has negligible H_2_O absorbability (Fig. 3a). Furthermore, soaking in neutral and acidic (0.1 M HCl) H_2_O for 2 d does not affect the powder X-ray diffraction (PXRD) patterns of **1** (Fig. 3b). These results show that **1** has outstanding stability and low water absorbability.

### Crystal structure of CO_2_-loaded soft crystal

Because soft crystal **1** retains its single crystalline nature after absorption of CO_2_, we were able to determine the structure of the CO_2_-loaded crystal **1**·2CO_2_ by using standard single-crystal X-ray diffraction analysis. The space group of **1**·2CO_2_ was found to be *C*2/*c*, which can be considered as being a transform to a *C*-centered lattice with space group *P*2/*n*. The coordination environments of the copper ion in **1** and **1**·2CO_2_ are similar (Figs. 2a and 2d), while the distance between the copper center and NTf_2_^‒^ anion is 7% shorter (Cu1′-O1′ = 2.600(2) Å) and that between the copper center and bpp ligands is slightly longer (Cu1′–N1′ = 2.015(3) Å, Cu1′–N2′ = 2.018(3) Å) in **1**·2CO_2_ compared to **1**. The toroidal shape of 1D chains in **1**·2CO_2_ are almost unchanged (6.3 Å × 12.3 Å, Fig. 2f and Supplementary Fig. 2) in comparison with **1**, and the dihedral angles of neighboring pyridine rings coordinated to the same copper ions decrease from 69°/68° in **1** to 59°/64° in **1**·2CO_2_. Moreover, **1**·2CO_2_ contains slightly different quasi 2D layers in comparison to **1**. Specifically, each 1D chain in **1**·2CO_2_ is inclined against the plane composed of copper ions, resulting in a slightly larger interchain distance (10.3 Å) (Supplementary Fig. 3). The interlayer space is occupied by both NTf_2_^‒^ anions and CO_2_ molecules, and the presence of absorbed CO_2_ causes a significant expansion of the interlayer distance from 8.0 to 8.6 Å (Supplementary Fig. 3). Consequently, the cell volume of **1**·2CO_2_ is 8.5 % larger than that of **1** largely because of a 7.5% expansion of the interlayer.

As mentioned above, all NTf_2_^‒^ anions in **1** are arranged in a parallel manner in the *trans* conformation. However, after CO_2_ absorption, six NTf_2_^‒^ anions exist in a *cis* conformation (Supplementary Fig. 1) and are positioned at corners of hexagonal vertexes (Supplementary Fig. 5) to form a cavity which encloses two CO_2_ molecules (Fig. 2e). The significant changes taking place in the interlayer distance and conformation of NTf_2_^‒^ anions, along with other slight structural changes suggest that the soft crystal **1** undergoes an induced-fit type change to accommodate CO_2_.

**Fig. 4:**
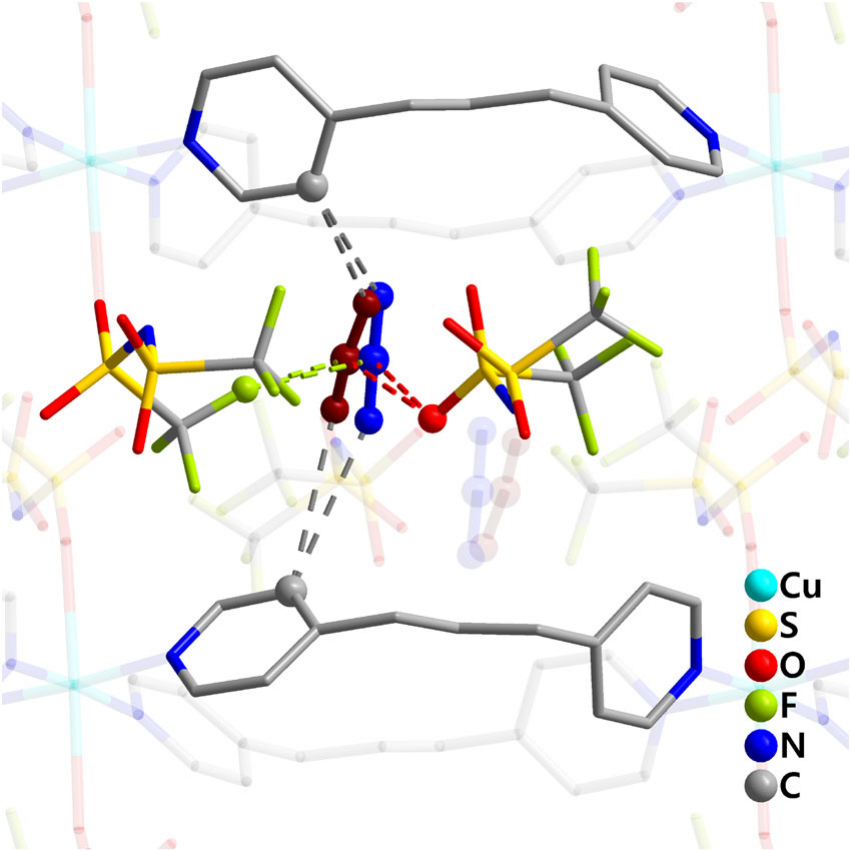
CO_2_-Absorbed structure. View of the intermolecular contacts between the CO_2_ molecule (blue: molecule A, red: molecule B) and structural components in **1**·2CO_2_.

Interactions that exists between the 1D chains and CO_2_ in **1**·2CO_2_ are shown in Fig. 4. CO_2_ molecules in **1**·2CO_2_ are disordered over two sites with occupancies of 0.66 (molecule A) and 0.34 (molecule B). The shortest contact exists between a fluorine of one NTf_2_^‒^ anion and the carbon of CO_2_ with F•••C distances of 2.95(2) (molecule A) and 2.68(5) Å (molecule B), which are shorter than the sums of their van der Waals radii (3.05–3.23 Å)^22^. On the other hand, the distances between oxygen of another NTf_2_^‒^ anion and the carbon of CO_2_ in molecule A (O•••C = 3.02(2) Å) and molecule B (O•••C = 3.28(5) Å) are comparable with the sums of their van der Waals radii (3.00–3.35 Å)^22^. Considering that molecule A has a larger occupancy, it is expected that both fluorine and oxygen in the NTf_2_^‒^ anion should play prominent roles in governing CO_2_ absorption. Weak hydrogen bonding interactions also exist between the bpp ligand and CO_2_ (H•••O and C•••O = 2.52(2) and 3.30(2) Å & 2.91(4) and 3.42(4) Å in molecule A and 2.58(4) and 3.34(4) Å & 2.83(6) and 3.34(6) Å in molecule B, Fig. 4).

The degree of quadrupole-quadrupole interactions between CO_2_ molecules is related to the distances between the carbons of two neighboring CO_2_ molecules. In **1**·2CO_2_, the respective distances between CO_2_ carbons in molecules A and A, molecules A and B, and molecules B and B are 3.82(3) Å, 4.03(6) Å and 4.28(8) Å. Although the crystal structure of **1**·2CO_2_ was determined at a temperature below the sublimation point of CO_2_, the intermolecular C–C distances are longer than the intermolecular distance (3.58 Å) in solid CO_2_^23^. Moreover, the C–C distance between CO_2_ molecules in molecules B and B is greater than 4.1 Å, which is the intermolecular C–C distance in gaseous CO_2_^24^. The differences in the intermolecular CO_2_•••CO_2_ distances show that a quadrupole-quadrupole interaction contributes little to stabilization of absorbed CO_2_ in **1**·2CO_2_ and, consequently, demonstrate that interactions with both a fluorine and oxygen atom in the NTf_2_^‒^ anion are the most influential in governing absorption of CO_2_ gas by **1**.

### Theoretical calculations

A density functional theory calculation was performed under periodic boundary conditions to gain more information about interactions occurring between NTf_2_^‒^ anions and CO_2_ in **1**·2CO_2_. The binding energy (*E*_b_) was determined by using the equation *E*_b_ = *E*_Framework+gas_ ‒ (*E*_Framework_ + *E*_Gas_), where *E*_Framework + gas_ and *E*_Framework_ are the respective energies of the complete complex **1**·2CO_2_ and only the framework of **1**·2CO_2_, and *E*_Gas_ is the energy of CO_2_ in a sufficiently large super cell. The calculated *E*_b_ value was found to be ‒31.3 kJ mol^−1^, which is consistent with the experimentally determined enthalpy of CO_2_ absorption. Next, an analysis of the interaction of **1**·2CO_2_ was performed, utilizing a cluster model and an energy decomposition analysis with the natural orbitals from chemical valence theory, in order to elucidate the nature and trend of the interaction that most strongly contributes to CO_2_ absorption. The results (Supplementary Table 4) show that large contributors to the stabilization of the structure of **1**·2CO_2_ are a dispersion force (‒28.7 kJ mol^−1^) and an electrostatic interaction (‒25.0 kJ mol^−1^), and that orbital interaction energy (‒13.3 kJ mol^−1^) is a minor contributor. This result is consistent with previous findings, which suggest that calculated Lewis acid-base binding energies between CO_2_ and naked anions are inversely proportional to the solubility of CO_2_ in ILs. Thus, Lewis acid-base interactions are not important contributors to solubilities of CO_2_ in ILs^25^.

## Discussion

**Fig. 5:**
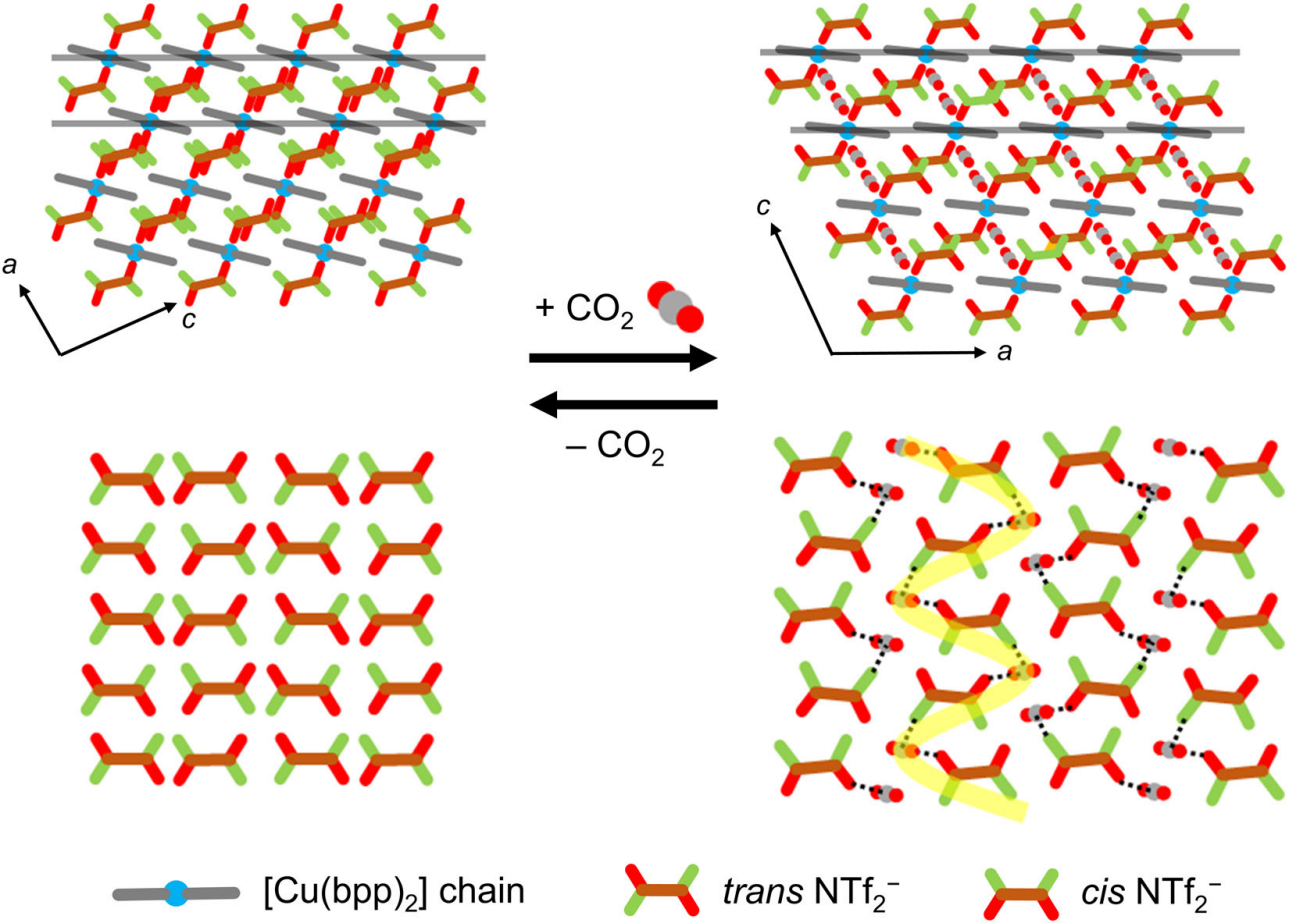
Schematic representation of CO_2_ absorption process. Schematic views of structural changes occurring in the CO_2_ absorption process by soft crystal **1**. The upper figures show packing structures viewed along the *b*-axis and bottom figures show arrangements of NTf_2_^‒^ anions in the presence and absence of CO_2_. The 1D [Cu(bpp)_2_] chains are oriented along the *b*-axis. The dotted lines indicate intermolecular interactions between the NTf_2_^‒^ anion and CO_2_. The yellow meandering line shows the 1D alternate arrangement of •••NTf_2_^‒^•••CO_2_•••NTf_2_^‒^•••CO_2_•••.

In Fig. 5 are displayed schematic views of the structures of soft crystal **1** formed during the CO_2_ absorption process. Analysis of the structures before and after CO_2_ absorption shows that the CO_2_ absorption process by **1** has features that are similar to those of ILs. Firstly, **1** preferably absorbed CO_2_ over other gases such as N_2_ and Ar. Secondly, during absorption, the lattice of **1** expands to create an interstitial space for CO_2_^26^, while the distance between the cation (in this case, copper) and anion (in this case, NTf_2_^‒^) changes only slightly^27^. In addition, our studies have uncovered a unique feature not observed previously in studies of CO_2_ absorption by ILs. Specifically, we obtained direct evidence showing that the NTf_2_^‒^ anion undergoes a conformational change from the *trans* to *cis* upon CO_2_ binding. In ILs, the NTf_2_^‒^ anion can exist in two possible conformations (*trans* and *cis*) at room temperature with the *trans* form being thermodynamically more stable^15,28^. The reason why the conformation of the NTf_2_^‒^ anion changes when **1** absorbs CO_2_ is that cohesion energy is enhanced owing to the larger dipole moment of the *cis* form (dipole moments for *trans* and *cis* forms are 0.2–0.4 and 4.4–5.4, respectively)^29^. This enables the absorbed CO_2_ molecule interact more strongly and cooperatively with the fluorine and oxygen atoms of the two neighboring *cis*- NTf_2_^‒^ anions by taking full advantage of delocalized negative charges (Supplementary Table 3), in the 1D alternate arrangement of •••NTf_2_^‒^•••CO_2_•••NTf_2_^‒^•••CO_2_•••.

Gas absorption taking place in association with a change between a closed structure and an open structure of an absorbent is dominated by a balance between the energy barrier for the structural transition of the absorbent and the enthalpy change associated with the open form absorbing the gas^30,31^. The fluorinated moieties contribute to decreasing the energy barrier for the structural transition because they have an ability to delocalize charge (Supplementary Table 3) and low electronic polarizability that leads to weakening intermolecular interactions^32^. The effect of the fluorinated groups on the gas absorption enthalpy change is likely not related to Lewis basicity because the result of this study reveal that Lewis acid-base interactions are not important contributors to solubilities of CO_2_. Indeed, the most basic sites in anions are close to the most acidic sites in cations, which prevents the occurrence of Lewis acid-base interactions between CO_2_ and the most basic sites. Although the issue requires more detailed studies, it appears that charge delocalization and low electric polarizability promoted by fluorination have respective impacts on electrostatic interactions and dispersion forces that influence CO_2_ absorption.

Prior to the study described above, numerous experiments have been conducted to elucidate the CO_2_ absorption states in ILs^7–12^. In this effort, using the soft crystal **1** as a surrogate, we were able to visualize interactions that occur between the NTf_2_^‒^ anion component of ILs and CO_2_. The pyridine rings in bpp ligands and NTf_2_^‒^ anions in **1** synergistically constrain CO_2_ molecules in the crystal. The NTf_2_^‒^ anion contains the primary absorption sites for CO_2_ comprised of a trifluoromethyl fluorine and sulfonyl oxygen atom. The bpp ligand supports the NTf_2_^‒^-CO_2_ interaction through the formation of a weak phenyl hydrogen-CO_2_ interaction. As a result, a dispersion force and electrostatic interaction are the main contributors to stabilization of the CO_2_-absorbed complex. Furthermore, a conformational change of the NTf_2_^‒^ anions from *trans* to *cis* occurring upon CO_2_ capture by **1** also contributes to the absorption capacity. Of course, we understand that the observations made in this investigation have been made using a surrogate and not under real IL conditions especially because of differences in temperature and fluidity of the system. In addition, ILs with the NTf_2_^‒^ anion are not practical for CO_2_ separation because the perfluorinated groups containing NTf_2_^‒^ anion is not an environment-friendly. Nevertheless, the findings should not only increase the level of understanding of CO_2_ absorption by ILs but also provide new approaches to probe interactions between ILs and CO_2_ and to design new systems for CO_2_ separation applications.

## Methods

### Synthesis

Commercially available reagents were used as received without further purification. Cu(NTf_2_)_2_ and Cu(NMes_2_)_2_ were synthesized using previously described procedures^28,33^.

A single-crystal of [Cu(NTf_2_)_2_(bpp)_2_] (**1**) was prepared by using a diffusion method in the straight glass tube containing a bottom layer of 0.1 M aqueous Cu(NTf_2_)_2_, a middle layer of 1:1 water and MeOH, and a top layer of 0.2 M bpp in MeOH. After 1 week, the formed transparent and rod like purple single crystals were separated by filtration, washed with water, and dried in the atmosphere. Elemental analysis was used to confirm the atomic composition and purity of the as-synthesized single crystals. Elemental analysis (%) calcd for C_30_H_28_Cu_1_F_12_N_6_O_8_S_4_: C 35.31, H 2.77, N 8.24, F 22.34, S 12.57. Found: C 34.86, H 2.57, N 8.04, F 22.77, S 12.69. The thermogravimetric curve and Fourier transform infrared spectrum of **1** are shown in Supplementary Figs. 7 and 8.

A single-crystal of {[Cu(NMes_2_)_2_(bpp)_2_]·3H_2_O} (**2**·3H_2_O) was prepared by using a solvent evaporation method in the beaker containing 0.1 M aqueous Cu(NMes_2_)_2_ and 0.2 M bpp in MeOH. After 1 week, the formed transparent and plate like blue single crystals were separated by filtration, washed with MeOH, and dried in the atmosphere. This crystal lost a part of hydrated water during drying, which was confirmed using elemental analysis. Elemental analysis (%) calcd for C_30_H_44.4_Cu_1_N_6_O_10.2_S_4_ (**2**·2.2H_2_O): C 42.69, H 5.30, N 9.96. Found: C 42.20, H 4.98, N 9.76. A single-crystal of [Cu(NMes_2_)_2_(bpp)_2_] (**2**) was prepared by drying **2**·3H_2_O under N_2_ flow at 373 K for 30 min.

### Single-crystal X-ray diffraction analysis of 1, 1·2CO_2_, and 2

Crystal data for **1** were collected at 173 K on a RIGAKU RAXIS-RAPID imaging-plate diffractometer with a graphite-monochromated Mo-Kα radiation (*λ* = 0.71075 Å). Crystal data for **2** were collected at 293 K on a RIGAKU XtaLab Synergy-R with a multi-mirror monochromated Mo-Kα radiation (*λ* = 0.71075 Å). The structures were solved by using direct method (SHELXT)^34^ and refined by using full-matrix least-squares techniques on *F*^2^ using SHELXL-2018^35^. All non-hydrogen atoms were refined anisotropically. Hydrogen atoms were located at geometrically calculated positions and refined using a riding model. Olex2 was used as a graphical user interface^36^. Crystallographic data are shown in Supplementary Tables 1 and 2 and Supplementary Data 1 and 3.

The structural determination of **1**·2CO_2_ was carried out using the following procedure. A single crystal of **1** was placed at the bottom of a glass capillary, which was then adhered to the base using glue. The base was installed on the absorption equipment (BELSORP-mini, MicrotracBEL Corp.) and the crystal was heated at 373 K for 30 min under a vacuum as the pre-treatment. After pre-treatment, CO_2_ gas at 40 kPa was added and the capillary was cut and sealed quickly. The capillary containing single crystal sample and CO_2_ gas was placed in the single-crystal X-ray diffractometer with a cryogenic unit, and cooled at a rate of 1 K min^−1^ from 293 K to 173 K, and then held at 173 K for 4 h. X-ray diffraction measurements were then made at 173 K on a RIGAKU XtaLAB P200 with a multi-mirror monochromated Cu-Kα radiation (*λ* = 1.5418 Å). Data analysis was performed in the manner described for **1**. Crystallographic data are shown in Supplementary Table 1 and Supplementary Data 2.

### Gas absorption isotherm experiments

CO_2_ (195 K), N_2_ (77 K and 195 K) and Ar (77 K and 195 K) absorption/desorption isotherms were measured by using a BELSORP-max (MicrotracBEL Corp.). Water absorption/desorption isotherms were measured at 298 K by using a BELSORP-aqua (MicrotracBEL Corp.). Before the measurement, the sample was heated at 373 K in a vacuum overnight for activation by BELPREP-vac (MicrotracBEL Corp.). High-pressure CO_2_ absorption/desorption isotherms were measured by using a BELSORP-HP (MicrotracBEL Corp.). Activation of the sample was performed on a BELSORP-HP at 373 K in a vacuum.

### Theoretical calculations

For determining the S–N bond rotational barrier in NTf_2_^‒^, relax scan calculations were performed using gaussian 09 Rev. E.01 software at the B3LYP/aug-cc-pVDZ level of theory^37^. A PBE functional^38^, with projector augmented wave potentials and van der Waals interaction corrected by using a D3 scheme^39^ to optimize the atomic positions of **1**, CO_2_ and **1**·2CO_2_, was used to obtain an estimate of the binding energy of CO_2_ (Supplementary Fig. 14). In all cases, spin polarized calculations were employed under periodic boundary condition and Γ-point approximation using the Vienna Ab initio Simulation Package (VASP)^40–43^ with the cut-off energy of 400 eV. The atomic charges were evaluated based on Bader analysis (Supplementary Table 3)^44–47^. In a manner that is similar to other reported approaches^48–50^, after optimization of **1**·2CO_2_, a model consisting of one CO_2_, three NTf_2_^−^ and three (4-pyridyl)butane, which are protonated instead of bonded to Cu^2+^ (Supplementary Fig. 15), was employed to investigate the nature of the interaction between CO_2_ and **1**. The positions of added H atoms were optimized with a PBE functional using Amsterdam Density Functional 2018 program^51^. A standard triple-ζ STO basis set with two sets of polarization functions (TZ2P)^52^ and a Grimme D3 type dispersion correction were applied to all atoms in the model. The model structure was optimized and analyzed by using the energy decomposition method^53^ combined with the natural orbitals for chemical valence theory^54,55^ with the same level of theory as that used for optimization of H atoms.

## Supplementary information

Supplementary Information

Supplementary Data 1

Supplementary Data 2

Supplementary Data 3

Description of Additional Supplementary Files

## Data Availability

Crystallographic data for the structures reported in this manuscript have been deposited at the Cambridge crystallographic Data Centre under deposition numbers CCDC 1981481 (**1**, Supplementary Data 1), 1981483 (**1**·2CO_2_, Supplementary Data 2), and 2021731 (**2**, Supplementary Data 3). Copies of the data can be obtained free of charge via https://www.ccdc.cam.ac.uk/structures/. All other relevant data that support the findings of this study are available within the manuscript and its Supplementary Information, or from the corresponding author upon reasonable request.
