# Supplementary material for: Understanding the interactions between the bis(trifluoromethylsulfonyl)imide anion and absorbed CO2 using X-ray diffraction analysis of a soft crystal surrogate
Source: Commun Chem. 2020 Oct 27;3:143. doi: 10.1038/s42004-020-00390-1 (PMC9814135; doi:10.1038/s42004-020-00390-1)
Supplement: Supplementary file 1 — Supplementary Information [file 42004_2020_390_MOESM1_ESM.pdf]

Supplementary Information

Understanding the interactions between the bis(trifluoromethylsulfonyl)imide anion and absorbed CO_2 using X-ray diffraction analysis of a soft crystal surrogate

Xin Zheng,[1] Katsuo Fukuhara,[1] Yuh Hijikata,[2] Jenny Pirillo,[2] Hiroyasu Sato,[3] Kiyonori Takahashi,[1,4] Shin-ichiro Noro[1,5*] and Takayoshi Nakamura[1,4*]

1 Graduate School of Environmental Science, Hokkaido University, Sapporo 060-0810, Japan.
2 Institute for Chemical Reaction Design and Discovery (WPI-ICReDD), Hokkaido University, Sapporo, 001-0021, Japan
3 Rigaku Corporation, Akishima, Tokyo 196-8666, Japan
4 Research Institute for Electronic Science, Hokkaido University, Sapporo 010-0020, Japan
5 Faculty of Environmental Earth Science, Hokkaido University, Sapporo 060-0810, Japan.

Table of contents

**1**	Methods	**2**
**2**	Crystal Structures	**3-10**
**3**	Thermogravimetric Analysis	**11**
**4**	Fourier Transform Infrared Spectroscopy	**12**
**5**	Adsorption/Desorption Isotherms	**13-16**
**6**	Theoretical Calculations	**17-20**

Supplementary Methods

1. Methods

Elemental analysis: Elemental analysis was measured in Global Facility Center, Hokkaido University, using MICRO CORDER JM10 (J-Science Lab), CE440 (Exeter Analytical), and DX-500 (Dionex).
Fourier Transform Infrared (FT-IR) spectrum: FT-IR spectrum was measured by using a Nicolet iS10 FT-IR (Thermo Scientific) at room temperature.
Thermogravimetric (TG) analysis: TG curve was measured by using a ThermoPlus2/TG-DTA8129 (Rigaku Corp.) from room temperature to 773 K under nitrogen flow of 100 mL/min and at a heating rate of 10 K/min.
Powder X-ray diffraction (PXRD) analysis: Powder X-ray diffraction analysis was measured by using a RINT-Ultima Ш diffractometer (Rigaku Corp.) with Cu-Kα radiation (λ = 1.5418 Å).

2. Crystal Structures

Supplementary Table 1. Crystallographic data of **1** and **1**·$2CO_2$.

	**1**	**1**·$2CO_2$
Chemical Formula	$[Cu(NTf_2)_2(bpp)_2]$	$\{[Cu(NTf_2)_2(bpp)_2]\cdot 2CO_2\}$
Formula	$C_{28}H_{28}CuF_6N_7O_4S_2$	$C_{30}H_{28}CuF_6N_7O_8S_2$
Formula weight	1020.36	1108.38
Crystal system	Monoclinic	Monoclinic
Space group	*P2/n*	*C2/c*
Temperature / K	173	173
a / Å	9.7230(9)	20.5810(6)
b / Å	12.1946(10)	12.3407(2)
c / Å	17.1072(14)	18.9377(6)
α / °	90	90
β / °	96.176(3)	114.545(4)
γ / °	90	90
V / Å^3	2016.6(3)	4375.2(2)
Z	2	4
GOF on F^2	1.102	1.280
R_1 $[I > 2\sigma(I)]^a$	0.0579	0.0823
R_w $[I > 2\sigma(I)]^b$	0.1530	0.2665

$^aR_1 = \sum||F_o| - |F_c||/\sum|F_o|$. $^bR_w = [(\sum w(|F_o|^2 - |F_c|^2)^2)/\sum w(F_o^2)^2]^{1/2}$.

Supplementary Table 2. Crystallographic data of **2**.

	**2**
Chemical Formula	$[Cu(NMes_2)_2(bpp)_2]$
Formula	$C_{30}H_{40}CuN_6O_8S_4$
Formula weight	804.46
Crystal system	Monoclinic
Space group	$P2_1/c$
Temperature / K	293
a / Å	9.7086(8)
b / Å	17.8811(11)
c / Å	11.4903(7)
α / °	90
β / °	109.910(8)
γ / °	90
V / Å^3	1875.5(2)
Z	2
GOF on F^2	1.282
R_1 [$I > 2\sigma(I)$][a]	0.1282
R_w [$I > 2\sigma(I)$][b]	0.2712

[a] $R_1 = \sum||F_o| - |F_c||/\sum|F_o|$. [b] $R_w = [(\sum w(|F_o|^2 - |F_c|^2)^2)/\sum w(F_o^2)^2]^{1/2}$.

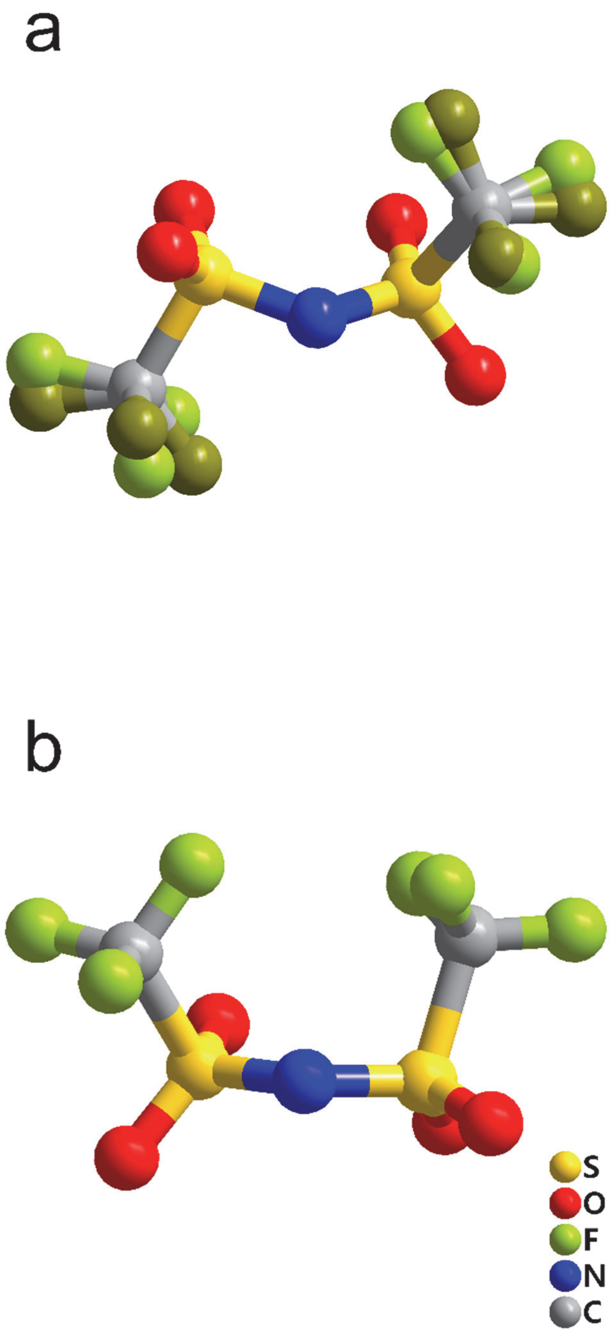

Supplementary Figure 1. Conformation of NTf_2^- anions in (a) **1** and (b) **1**·$2CO_2$. The CF_3 parts in **1** are disordered over two sites with occupancies of 0.66 and 0.34.

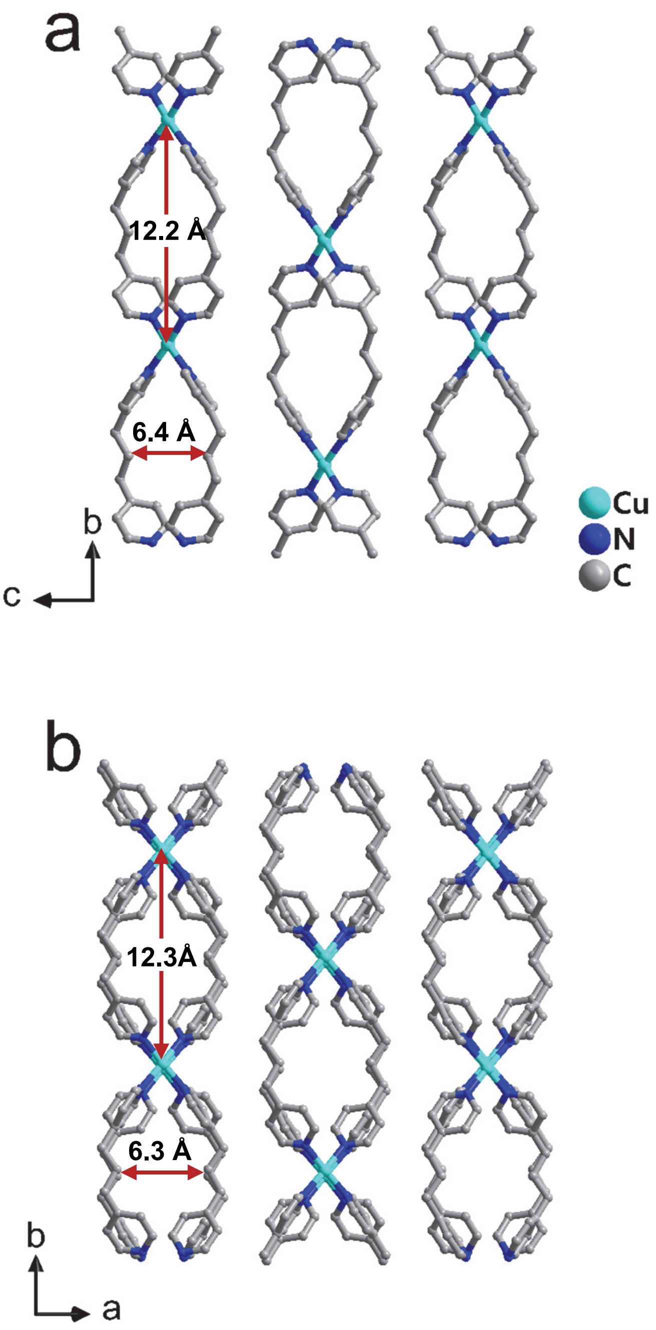

Supplementary Figure 2. One-dimensional structure of (a) **1** and (b) **1**·2CO_2 viewed along the *a* and *c* axes, respectively. The hydrogen atoms are omitted for clarity.

.

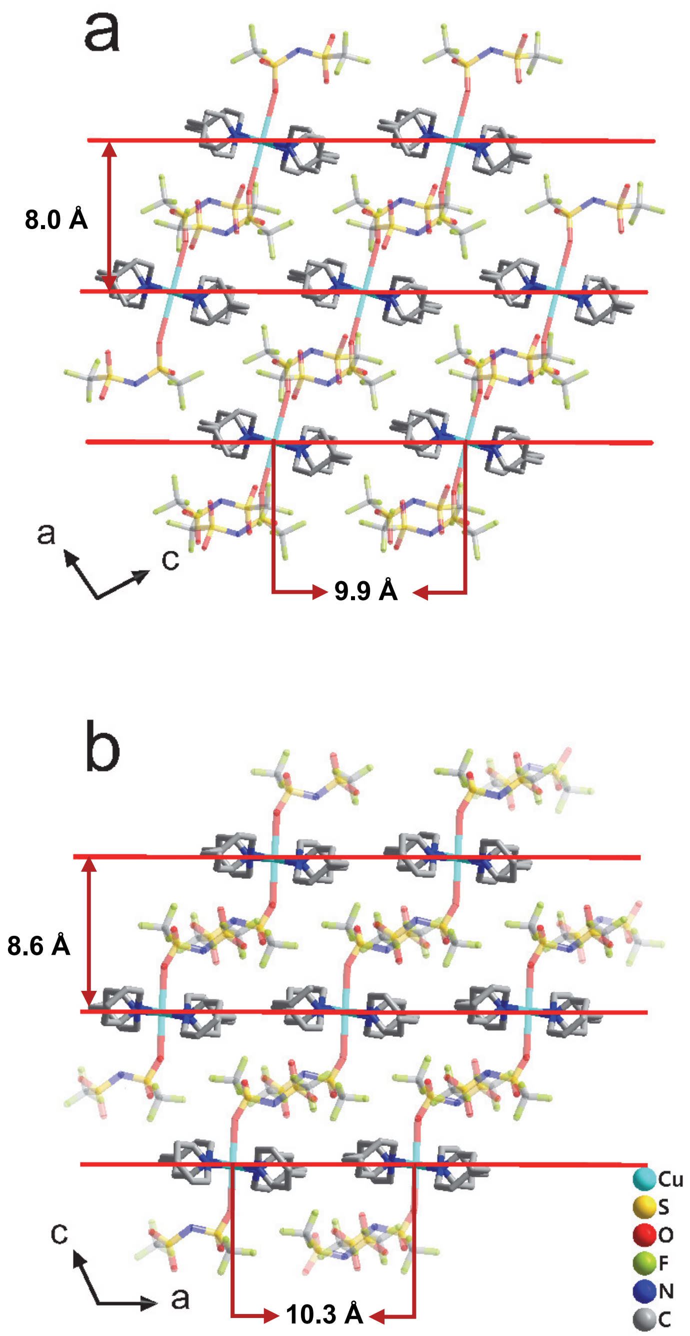

Supplementary Figure 3. Packing structures of (a) **1** and (b) **1**·2CO_2 viewed along the *b* and *b* axes, respectively. The hydrogen atoms and CO_2 molecules are omitted for clarity.

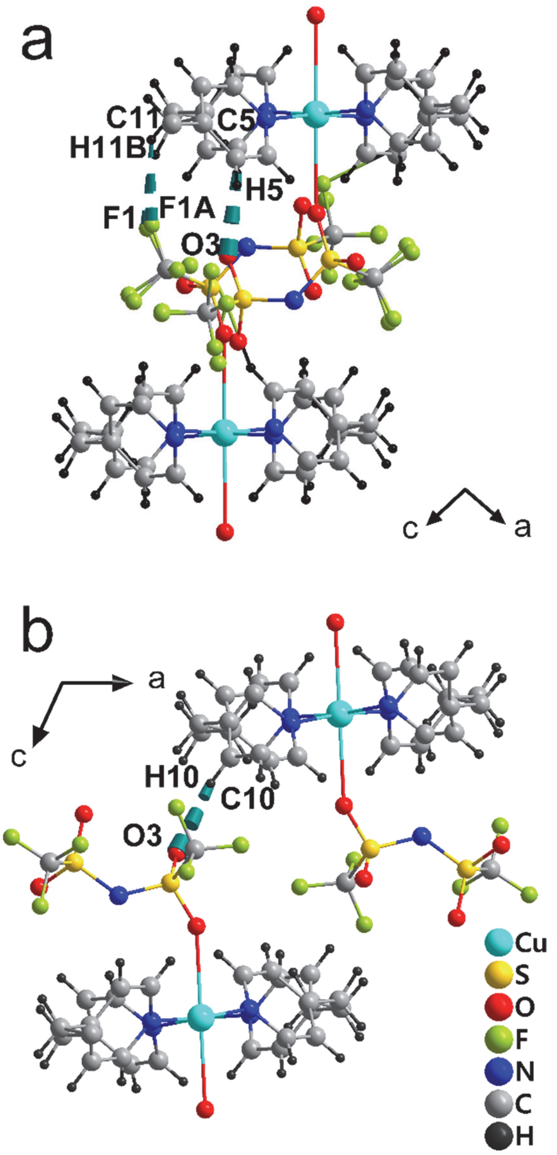

Supplementary Figure 4. View of the interchain interactions of (a) **1** and (b) **1**·$2CO_2$. The CF_3 parts in **1** are disordered over two sites with occupancies of 0.66 and 0.34. In **1**, there are two kinds of weak interactions; one is observed between the NTf_2^- fluorine atom and the bpp methylene hydrogen atom with F1•••H11B = 2.54(2) Å, F1•••C11 = 3.37(2) Å, and F1•••H11B-C11 = 140.9(5) °, F1A•••H11B = 2.43(2) Å, F1•••C11 = 3.24(3) Å, and F1•••H11B-C11 = 139.1(7) °. The other is between the NTf_2^- oxygen atom and the bpp pyridine hydrogen atom with O3•••H5 = 2.362(4) Å, O3•••C5 = 3.188(5) Å, and O3•••H5-C5 = 145.0(2) °. In **1**·$2CO_2$, one kind of interchain interaction is found between the NTf_2^- oxygen atom and the bpp pyridine hydrogen atom with O3•••H10 = 2.575(3) Å, O3•••C10 =3.359(5) Å, and O3•••H10-C10 = 142.3(2) °.

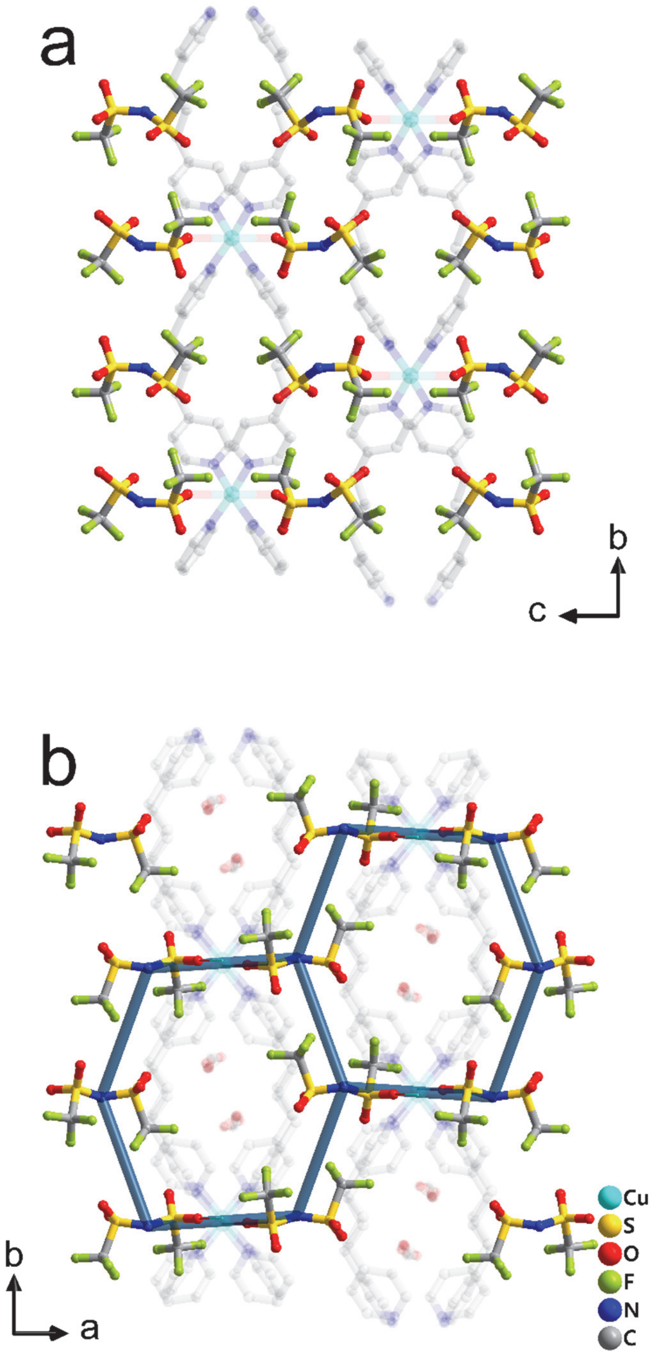

Supplementary Figure 5. Arrangement of NTf_2^- anions in (a) **1** and (b) **1**·$2CO_2$ viewed along the *a* and *c* axes, respectively. The hydrogen atoms are omitted for clarity.

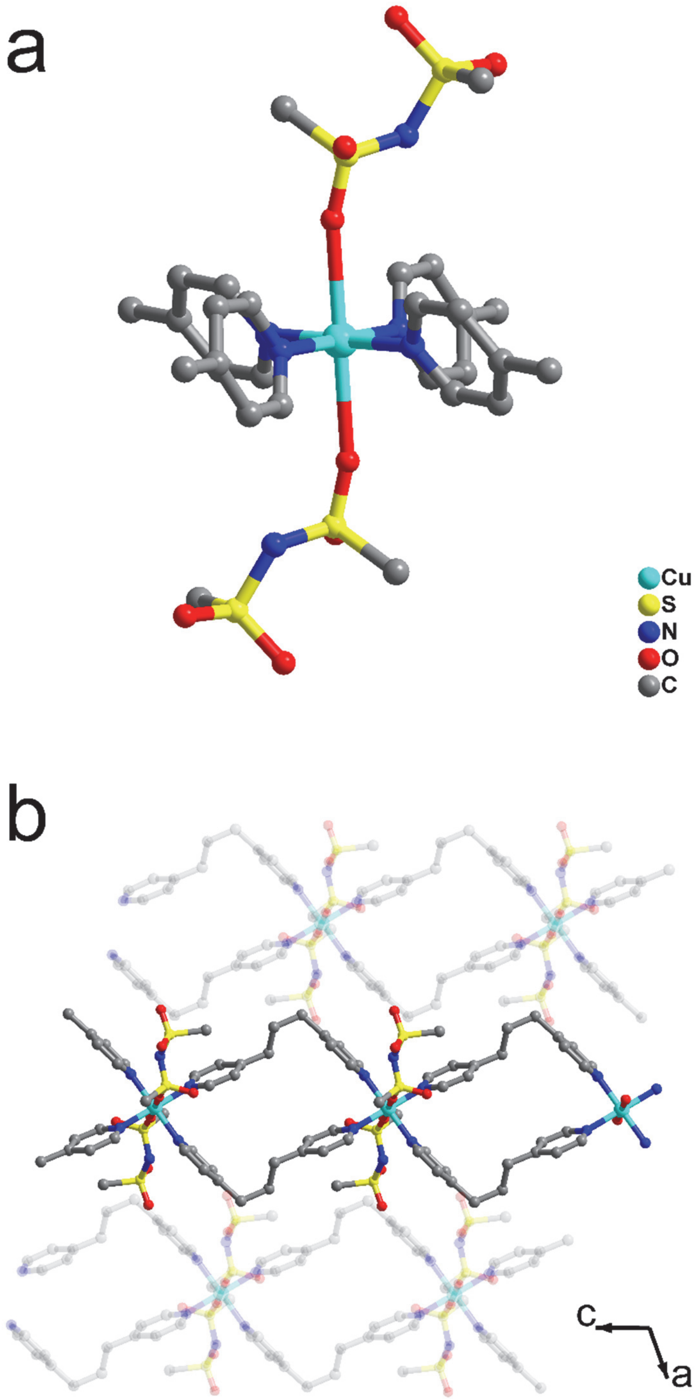

Supplementary Figure 6. Views of the crystal structure of **2**. (a) Coordination environment around the Cu center and (b) 1D chain structure.

3. Thermogravimetric Analysis

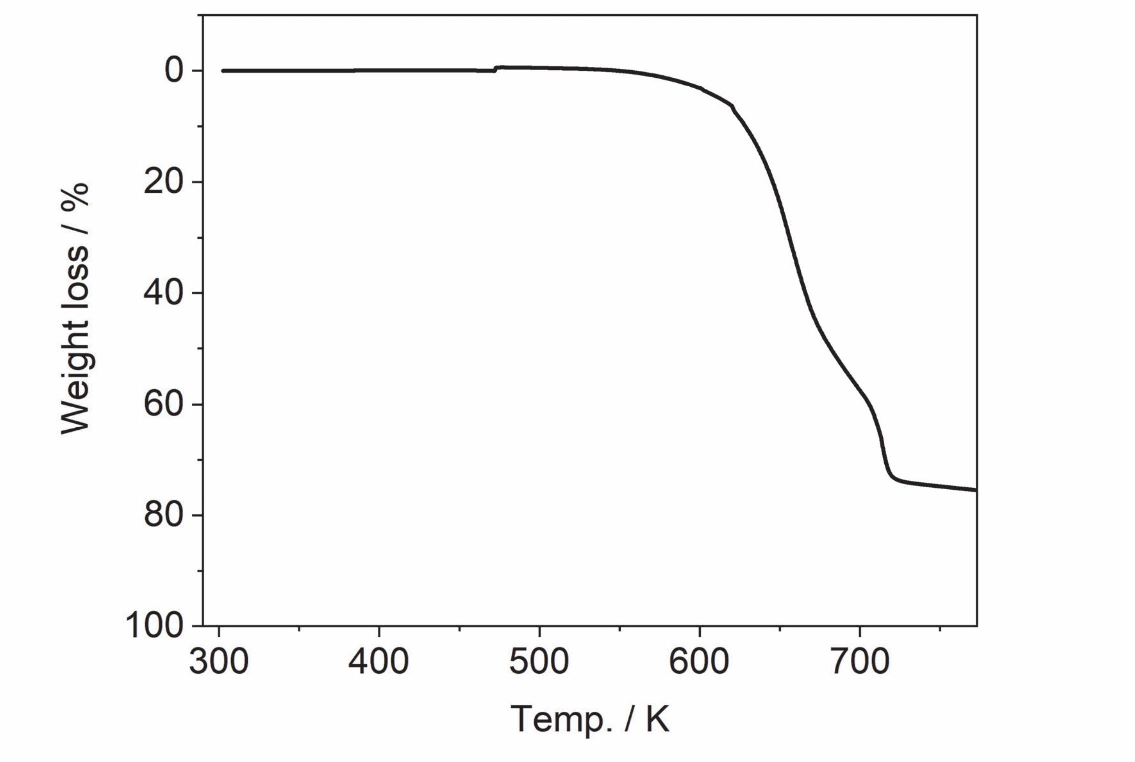

Supplementary Figure 7. TG curve of **1**. There is no weight loss until ca. 570 K, implying the thermal stability of **1**.

4. Fourier Transform Infrared Spectroscopy

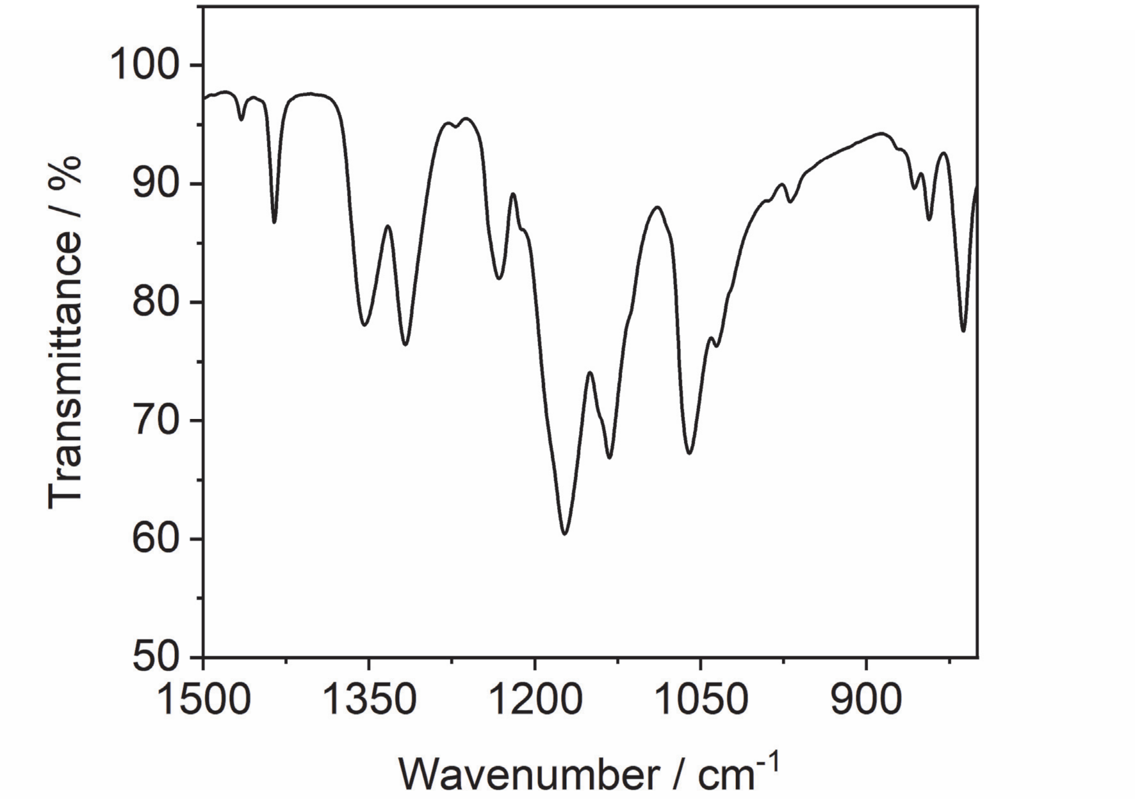

Supplementary Figure 8. FT-IR spectrum of **1**. The bands at 1317 and 1354 cm^{-1} can be assigned to the asymmetric vibration bands of the NTf_2^- sulfonyl group, while the band at 1132 cm^{-1} can be assigned to the symmetric vibration band of the NTf_2^- sulfonyl group. The band at 1173 cm^{-1} can be assigned to the vibration band of the NTf_2^- trifluoromethyl group.

5. Absorption/Desorption Isotherms

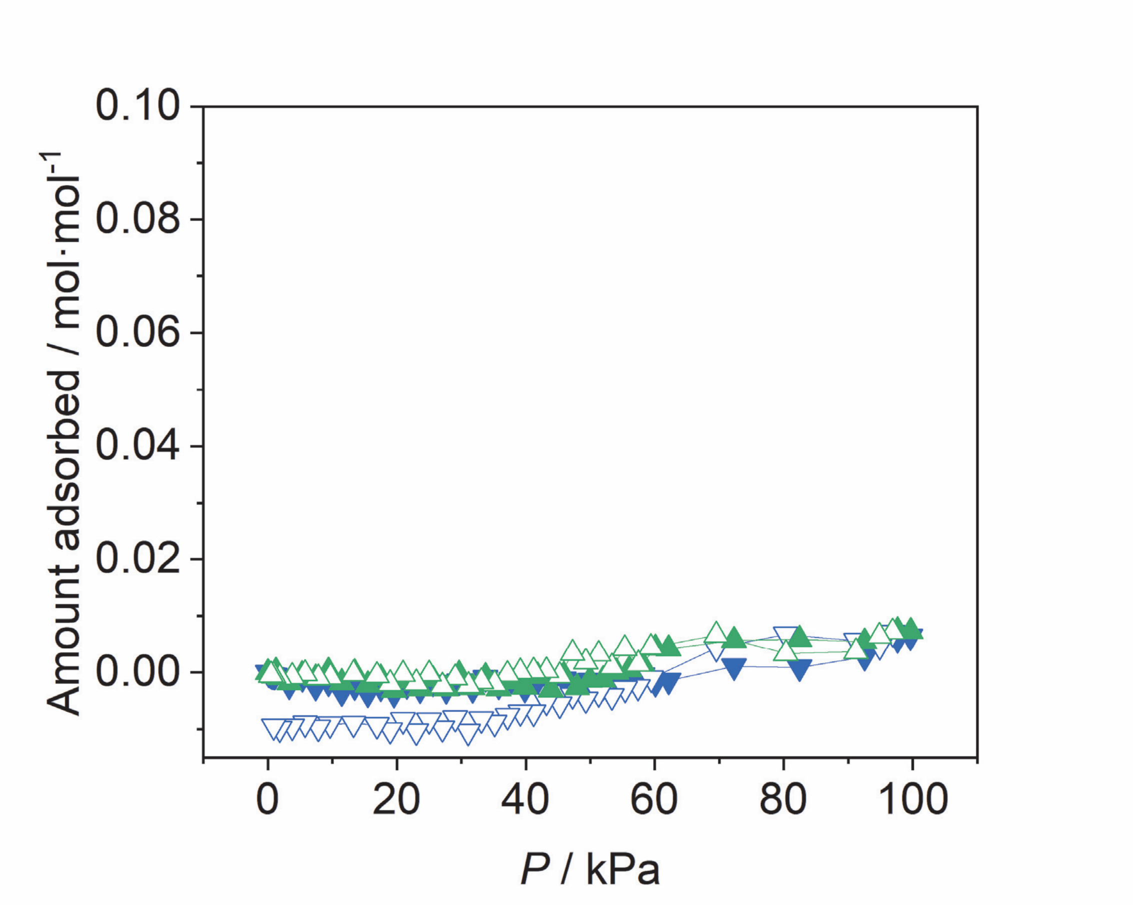

Supplementary Figure 9. Adsorption (closed symbols)/desorption (open symbols) isotherms of **1** for N_2 (blue reversed triangle) and Ar (green triangle) at 195 K.

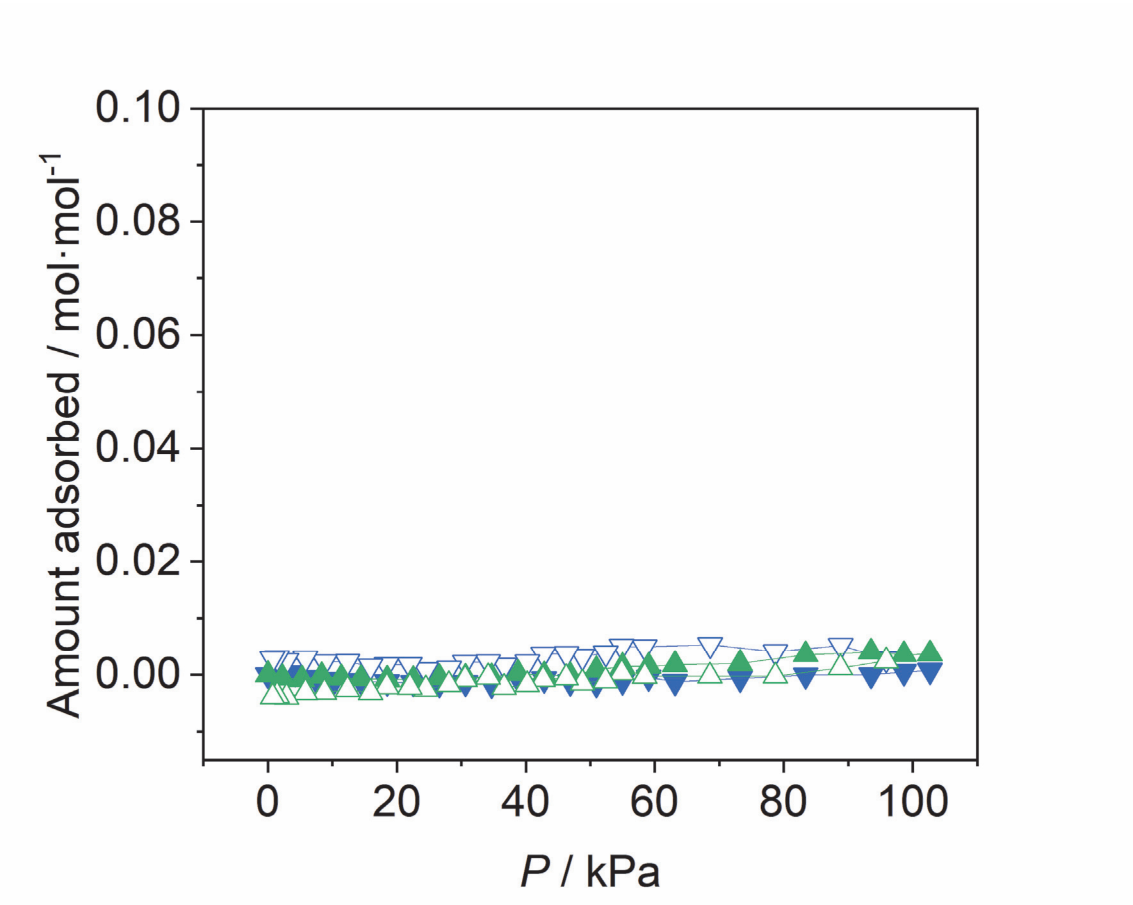

Supplementary Figure 10. Gas absorption (closed symbols)/desorption (open symbols) isotherms of **2** for N_2 (blue reversed triangle) and Ar (green triangle) at 195 K.

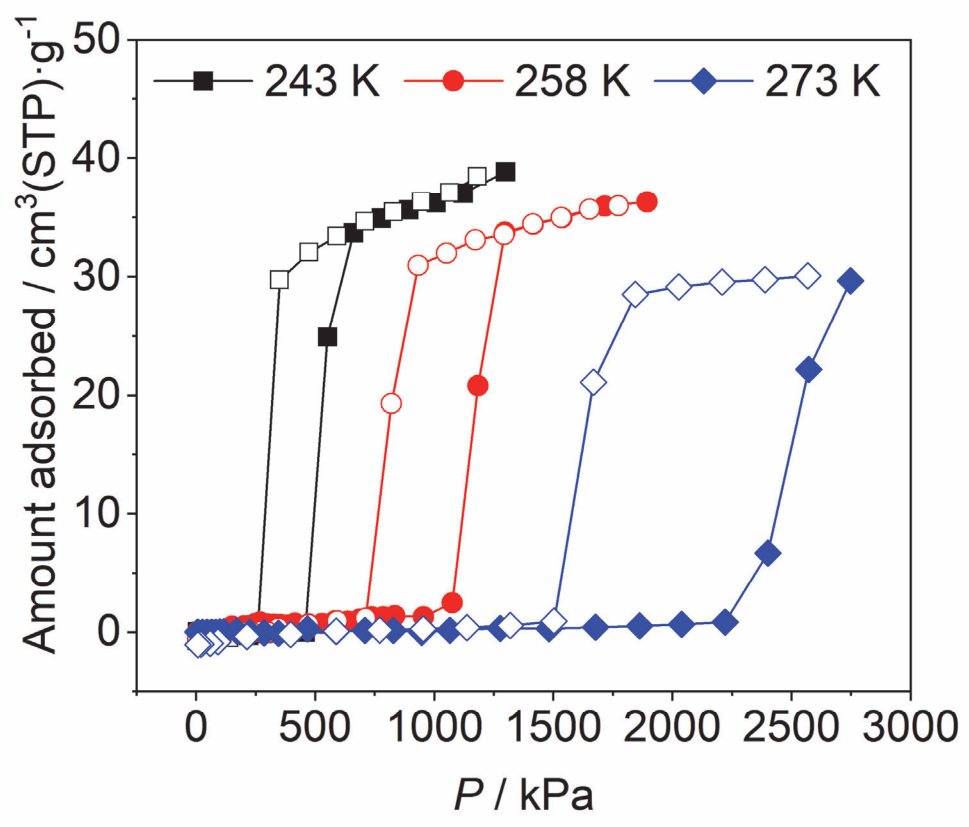

Supplementary Figure 11. CO_2 absorption (closed symbols)/desorption (open symbols) isotherms of **1** at 243 K (black square), 258 K (red circle), and 273 K (blue rhombus).

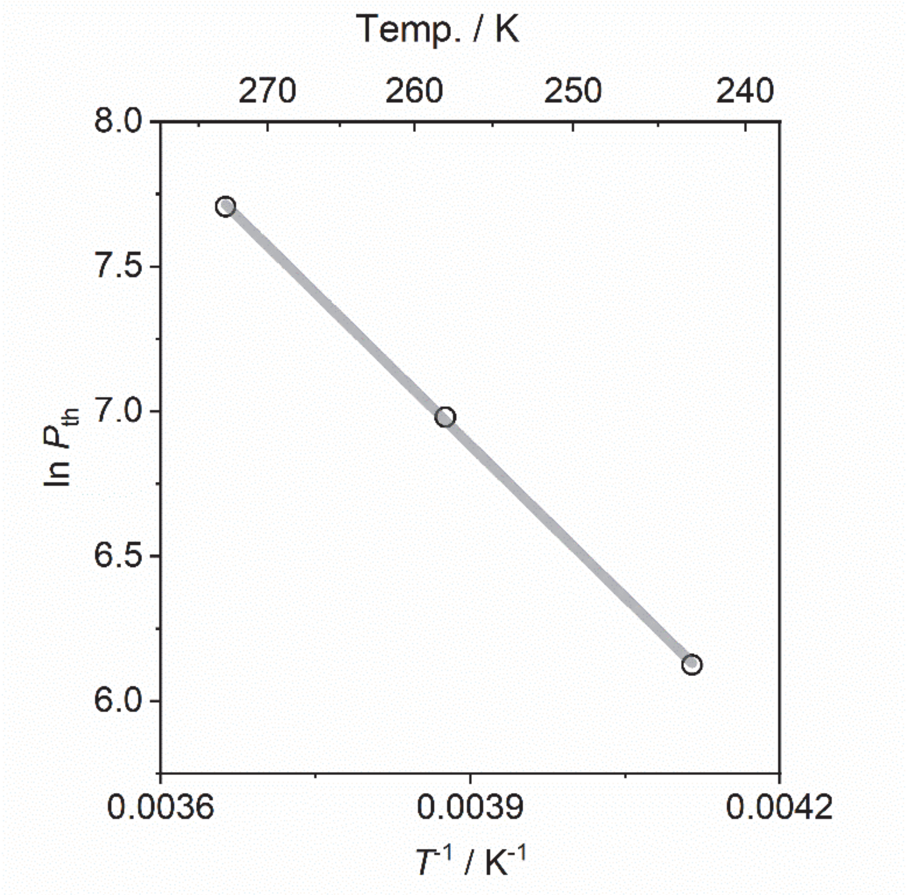

Supplementary Figure 12. Plot of lnP_{th} (P_{th} = the threshold pressures) against T^{-1}. From the CO_2 absorption isotherms at different temperatures (Figure S8), remarkable P_{th} are determined and they are regarded as the equilibrium pressures for the CO_2 absorption reaction. Therefore, the enthalpy of CO_2 absorption can be calculated using the following Clausius-Clapeyron equation,

$$\frac{Q_{\mathrm{st}}}{R} = \frac{d \ln P_{\mathrm{th}}}{\mathrm{d}T^{-1}}$$

where R and Q_{st} represent the gas constant and the enthalpy of absorption, respectively. The plot of ln P_{th} versus T^{-1} yields the straight line, whose slope affords the Q_{st} = –29.1 kJ mol^{-1}.

6. Theoretical Calculation

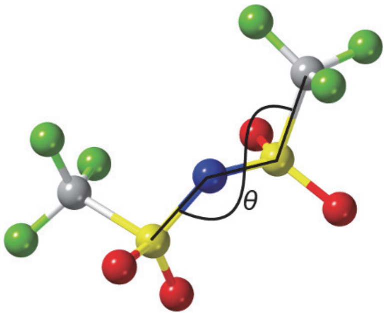

Supplementary Figure 13. Definition of the NTf_2^- dihedral angle for relax scan calculation.

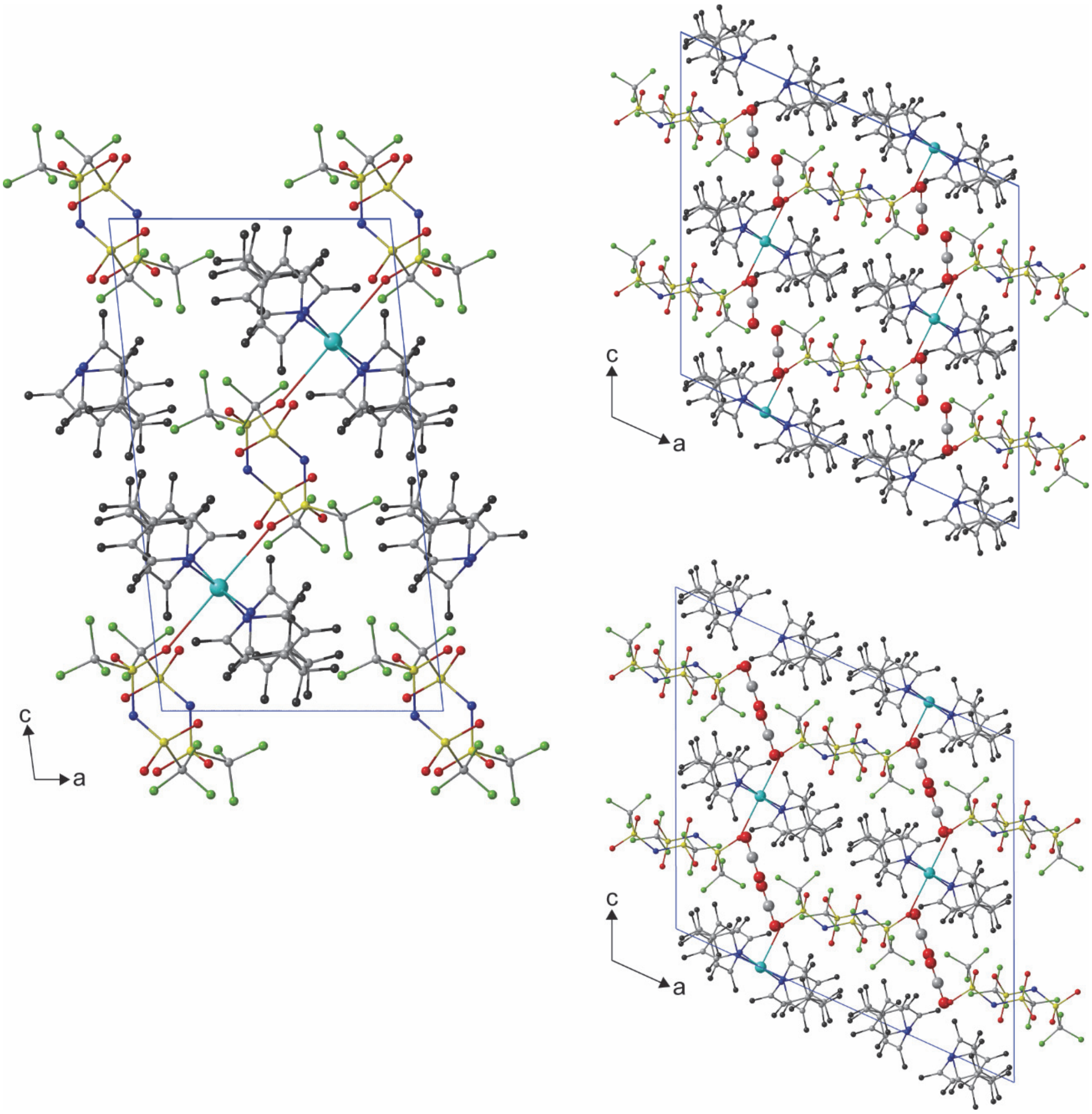

Supplementary Figure 14. Optimized structures of **1** (left) and **1**·2CO_2 (right). The upper and bottom right figures show the optimized structures with the disordered CO_2 molecules A and B, respectively. The binding energies, E_b, were calculated from these optimized structures to be –31.3 kJ·mol^{-1} for both disordered CO_2 molecules.

Supplementary Table 3. Atomic charges obtained from the Bader analysis for NTf_2^- anions in the model structure with CO_2 constructed from the CO_2-absorbed phase (**1**·$2CO_2$). The atom numbering figures are shown in the bottom. Atomic charges in the similar model without CO_2 constructed from the desolvated phase (**1**) are shown in parentheses.

**NTf_2^-** in the model		
F1: -0.607 (-0.608)	F2: -0.605 (-0.618)	F3: -0.598 (-0.603)
F4: -0.610 (-0.600)	F5: -0.611 (-0.608)	F6: -0.616 (-0.584)
O3: -1.260 (-1.261)	O4: -1.267 (-1.281)	O5: -1.264 (-1.278)
O6: -1.260 (-1.285)	S1: +2.992 (+3.027)	S2: +3.014 (+3.053)
N1: -1.538 (-1.577)	C2: +1.670 (+1.645)	C3: +1.645 (+1.600)
F1′: -0.602 (-0.603)	F2′: -0.612 (-0.618)	F3′: -0.601 (-0.608)
F4′: -0.610 (-0.608)	F5′: -0.603 (-0.584)	F6′: -0.615 (-0.600)
O3′: -1.274 (-1.281)	O4′: -1.252 (-1.261)	O5′: -1.271 (-1.278)
O6′: -1.291 (-1.285)	S1′: +2.970 (+3.027)	S2′: +3.004 (+3.053)
N1′: -1.518 (-1.577)	C2′: +1.657 (+1.645)	C3′: +1.664 (+1.600)

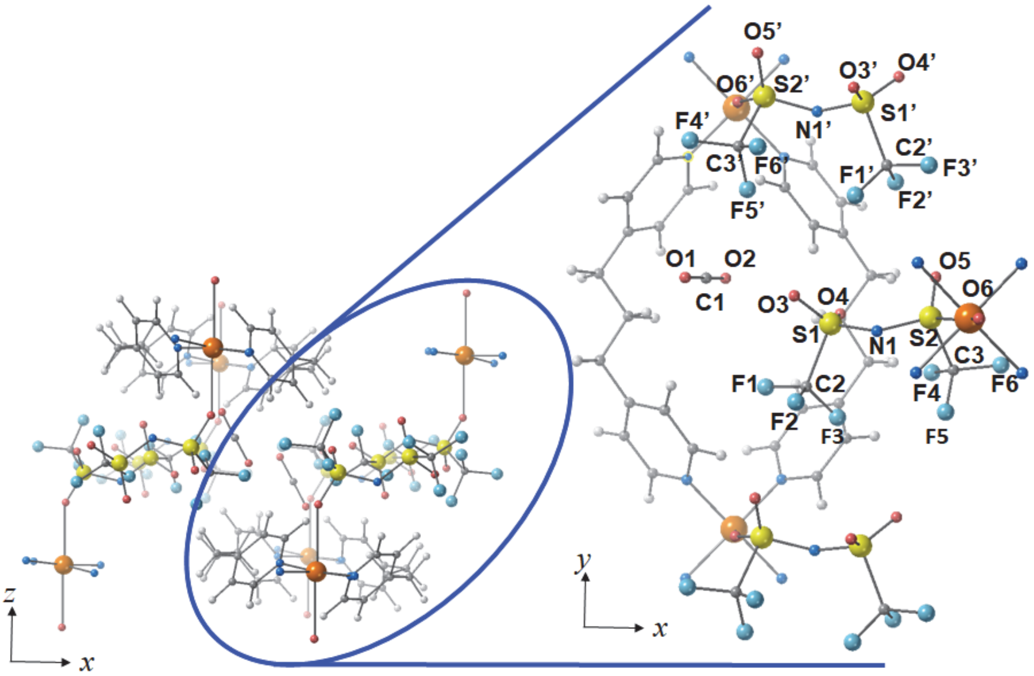

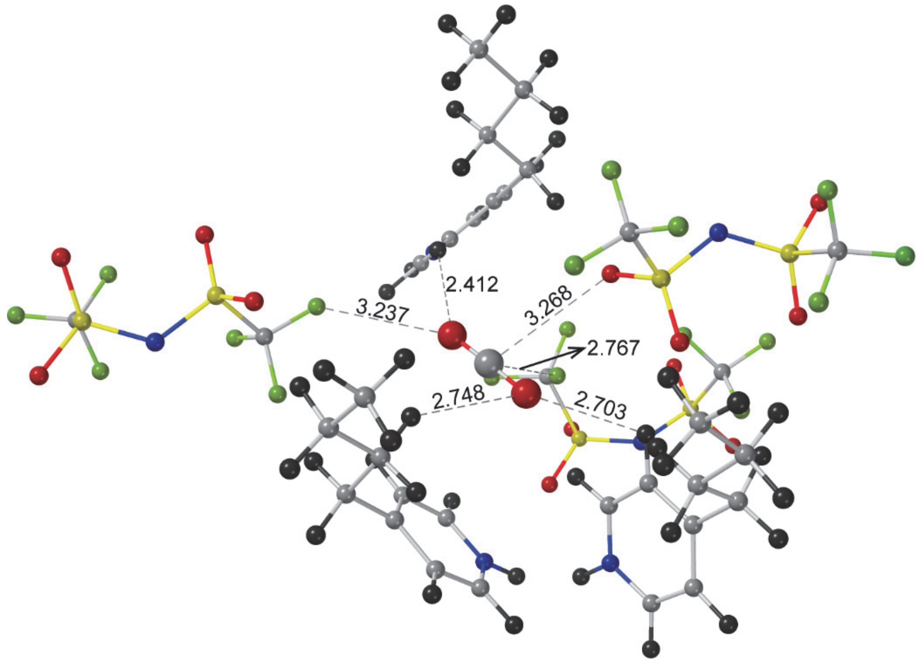

Supplementary Figure 15. Model structure used for the energy decomposition analysis with the natural orbitals from chemical valence theory (EDA-NOCV). The values indicate the distances (Å) between neighboring atoms.

Supplementary Table 4. Energetic components of interaction energy (in kJ mol^{-1}) in the framework•••CO_2 model structure used for the EDA-NOCV analysis.

E_{pauli}	31.1
E_{elst}	-25.0
E_{orb}	-13.3
E_{disp}	-28.7
E_{int}[a]	-35.9

[a] E_{int} is a summation of E_{pauli}, E_{elst}, E_{orb}, and E_{disp}.
