# Supplementary material for: Understanding the interactions between the bis(trifluoromethylsulfonyl)imide anion and absorbed CO2 using X-ray diffraction analysis of a soft crystal surrogate
Source: Commun Chem. 2020 Oct 27;3:143. doi: 10.1038/s42004-020-00390-1 (PMC9814135; doi:10.1038/s42004-020-00390-1)
Supplement: Supplementary file 5 — Description of Additional Supplementary Files [file 42004_2020_390_MOESM5_ESM.pdf]

Description of Additional Supplementary Files

File Name: Supplementary Data 1
Description: cif file of 1

File Name: Supplementary Data 2
Description: cif file of 1·2CO_2

File Name: Supplementary Data 3
Description: cif file of 2
